# Deep Brain Stimulation for Obsessive Compulsive Disorder: Evolution of Surgical Stimulation Target Parallels Changing Model of Dysfunctional Brain Circuits

**DOI:** 10.3389/fnins.2018.00998

**Published:** 2019-01-08

**Authors:** Patrick J. Karas, Sungho Lee, Joohi Jimenez-Shahed, Wayne K. Goodman, Ashwin Viswanathan, Sameer A. Sheth

**Affiliations:** Baylor College of Medicine, Houston, TX, United States

**Keywords:** DBS, OCD, review, surgical procedures, operative, brain circuitry

## Abstract

Obsessive compulsive disorder (OCD) is a common, disabling psychiatric disease characterized by persistent, intrusive thoughts and ritualistic, repetitive behaviors. Deep brain stimulation (DBS) is thought to alleviate OCD symptoms by modulating underlying disturbances in normal cortico-striato-thalamo-cortical (CSTC) circuitry. Stimulation of the ventral portion of the anterior limb of the internal capsule (ALIC) and underlying ventral striatum (“ventral capsule/ventral striatum” or “VC/VS” target) received U.S. FDA approval in 2009 for patients with severe, treatment-refractory OCD. Over the decades, DBS surgical outcome studies have led to an evolution in the electrical stimulation target. In parallel, advancements in neuroimaging techniques have allowed investigators to better visualize and define CSTC circuits underlying the pathophysiology of OCD. A critical analysis of these new data suggests that the therapeutic mechanism of DBS for OCD likely involves neuromodulation of a widespread cortical/subcortical network, accessible by targeting fiber bundles in the ventral ALIC that connect broad network regions. Future studies will include advances in structural and functional imaging, analysis of physiological recordings, and utilization of next-generation DBS devices. These tools will enable patient-specific optimization of DBS therapy, which will hopefully further improve outcomes.

## Pathophysiology of Obsessive-Compulsive Disorder (OCD)

Obsessive-compulsive disorder (OCD) is characterized by repetitive, intrusive, and persistent thoughts (obsessions) and behaviors (compulsions), present in 2–3% of the general population (Goodman et al., 2014). The majority of patients with OCD respond to pharmacotherapy and cognitive-behavioral therapy, but as many as 10–20% of patients fail to improve (Denys, 2006). In the past half century, these refractory cases have been treated with ablative surgeries, such as anterior capsulotomy and cingulotomy, with meaningful improvement seen in 30–70% of the patients (Ballantine et al., 1987; Oliver et al., 2003; Sheth et al., 2013). Given the success of deep brain stimulation (DBS) in surgery for movement disorders such as Parkinson’s disease and essential tremor, this therapy has also been applied to OCD over the past few decades. DBS offers certain advantages over lesioning methods, including its adjustable and reversible nature. The ability to turn stimulation on and off also facilitates the design of randomized, sham-controlled studies. Disadvantages include the need for patients to remain “tethered” to clinical sites experienced with DBS programming for OCD, as well as the need for a permanent implant and associated hardware-related inconveniences and potential complications.

The severity of OCD symptoms is measured using the Yale-Brown Obsessive-Compulsive Scale (Y-BOCS) that assigns up to 20 points for obsessive symptoms and 20 points for compulsive symptoms (Goodman et al., 1989). Patients with Y-BOCS scores greater than 24 (out of a possible 40) are considered to have severe OCD. Treatment response to pharmaceutical, psychotherapeutic, or surgical therapies is generally defined as a reduction of 35% or more in Y-BOCS, a threshold that has been used historically both in pharmaceutical trials and surgical trials for DBS.

**FIGURE 1:**
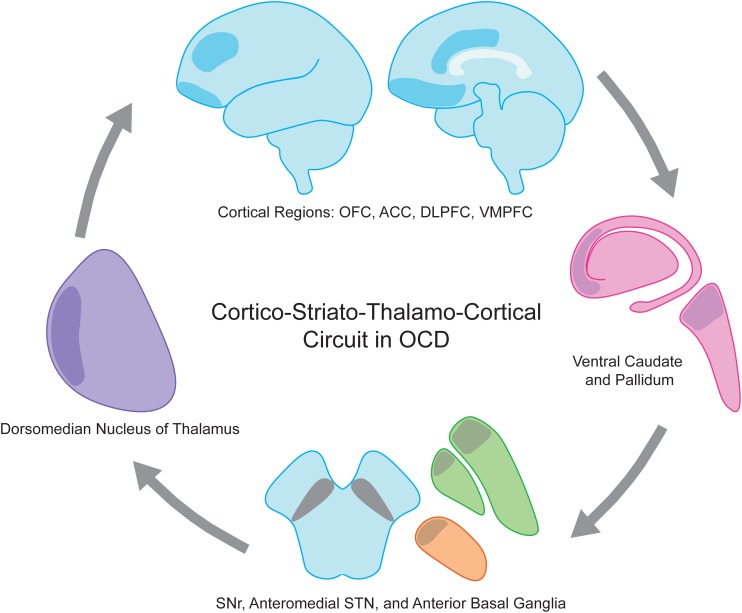
Obsessive compulsive disorder (OCD) is a disease of the cortico-striato-thalamo-cortical circuit. Involved anatomic regions include executive and limbic cortical regions such as OFC, ACC, DLPFC, and VMPFC (shaded blue), ventral caudate and pallidum (shaded pink), anterior globus pallidus interna and externa (shaded green), anteriomedial STN (shaded orange), SNr, and dorsomedian nucleus of the thalamus (shaded purple). ACC, anterior cingulate cortex; DLPFC, dorsolateral prefrontal cortex; OFC, orbitofrontal cortex; SNr, substantia nigra pars reticulata; STN, subthalamic nucleus; VMPFC, ventromedial prefrontal cortex.

**FIGURE 2:**
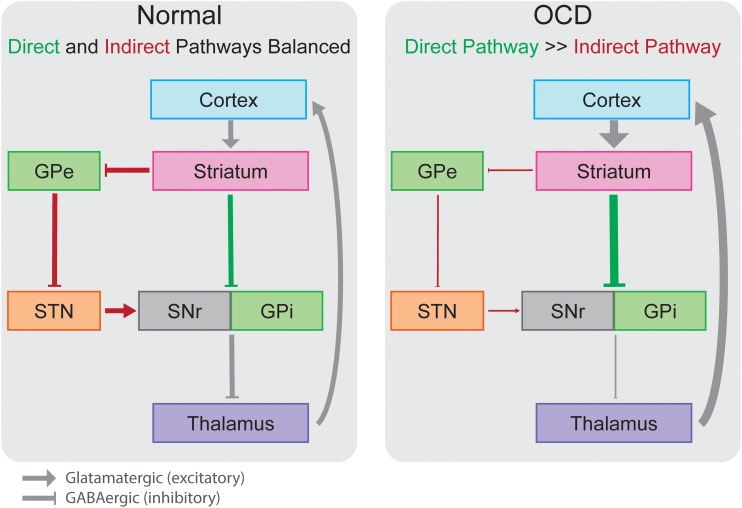
In the normally functioning cortico-striato-thalamo-cortical circuit, direct (green) and indirect (red) pathways lead to increased or decreased inhibition of the thalamus, respectively, in a balanced manner. In OCD, overactivation of the direct pathway out of proportion to the indirect pathway leads pathologic overactivity of cortical regions in an insidious excitatory loop. Boxes colored as in Figure 1 representing anatomic regions. GPe, globus pallidus externa; GPi, globus pallidus interna; SNr, substantia nigra pars reticulata; STN, subthalamic nucleus.

The pathophysiology of OCD involves many distinct cortical and subcortical regions. As for movement disorders, cortico-striato-thalamo-cortical (CSTC) loops have been employed as a useful framework for understanding the ability to modulate such broad cortical topography with focal stimulation in OCD (Pauls et al., 2014). Multiple CSTC loops are thought to be dysfunctional in OCD, spanning cortical regions including orbitofrontal (OFC), dorsolateral prefrontal (dlPFC), dorsal anterior cingulate (dACC), ventromedial prefrontal (vmPFC) and associated basal ganglia, thalamic, and limbic structures (Figure 1). CSTC loops contain adjacent but largely distinct subcortical direct and indirect pathways that ultimately lead to excitatory and inhibitory feedback, respectively, to cortical regions. Normally, glutamatergic projections from the cortex stimulate the striatum, where direct and the indirect pathways then emerge. In the direct pathway, activation of the striatum increases inhibitory GABAergic stimulation of the globus pallidus interna (GPi) and substantia nigra pars reticulata (SNr), which in turn decreases the inhibitory GABAergic output from GPi and SNr to the thalamus (Figure 2). As the thalamus sends excitatory glutamatergic stimulation back to cortical regions, the net effect of the direct pathway is a positive CSTC feedback loop. The indirect pathway on the other hand is a net inhibitory CSTC feedback loop. Indirect pathway GABAergic projections from the striatum to the globus pallidus externa (GPe) decrease the GPe’s GABAergic inhibition of the subthalamic nucleus (STN). Disinhibition of the STN allows for increased excitatory signaling to GPi and SNr, leading to net inhibition of the thalamus and its excitatory output to cortical regions.

Cortico-striato-thalamo-cortical loops have a parallel organization, involving distinct but at times partially overlapping regions of the cortex and nuclei of the striatum and thalamus. Two prefrontal CSTC loops, often combined and labeled the executive/associative circuit, are of particular interest in OCD: the lateral orbitofrontal circuit and the dorsolateral prefrontal circuit (Alexander et al., 1986). Originally, the connections between OFC, ventromedial caudate, medial dorsomedial GPi, rostromedial SNr, and dorsomedian (DM) nucleus of the thalamus that form the lateral orbitofrontal CSTC loop received the most attention due to converging evidence from human and animal studies.

Functional MRI studies in OCD patients have demonstrated aberrant connections between ventral striatum (VS) and OFC that correlate with illness severity (Beucke et al., 2013), whereas the activity in these regions decreases in response to first-line OCD treatments including selective serotonin reuptake inhibitors (SSRIs) and cognitive behavioral therapy (Nakao et al., 2005). In addition, single cell recordings of medium spiny neurons in the caudate that project to OFC demonstrated high firing rates during expression of OCD symptoms compared to resting state conditions (Guehl et al., 2008). Moreover, selective, repeated optogenetic stimulation of the excitatory OFC-VS projections led to OCD-like behaviors in mice such as excessive self-grooming (Ahmari et al., 2013). Taken together, these findings suggest that preferential activation of the orbitofrontal CSTC loop’s net excitatory direct pathway, out of proportion to the inhibitory indirect pathway, is a major contributor to OCD pathophysiology. The orbitofrontal CSTC loop is implicated in motor response to emotional stimuli; thus it has been hypothesized that this overactivation drives the compulsive and ritualistic features typical of OCD (Saxena and Rauch, 2000).

Hypoactivation of the dlPFC and dorsolateral caudate has also been observed in OCD (Levine et al., 1998), implicating the second prefrontal CSTC loop – the dorsolateral prefrontal circuit. The dorsolateral prefrontal loop involves dlPFC, dorsolateral caudate, lateral dorsomedial GPi, rostrolateral SNr, and the magnocellular and parvocellular components of the ventral anterior thalamus (Alexander et al., 1986). Hypoactivity of this circuit (i.e., overactivation of the indirect pathway) may contribute to the inability to switch between tasks that is characteristic of OCD (van den Heuvel et al., 2010).

Whereas the CTSC hypothesis provides a useful framework, a full appreciation of the pathophysiology of OCD is likely more complicated. While the classic CSTC model has described adjacent but distinct subcortical regions of the striatum, there is in fact overlap between regions allowing for overlapping input from cortical regions belonging to different CSTC loops (Draganski et al., 2008; Haber and Knutson, 2010). The overlap and convergence of cortical projections from distinct classical CSTC loops has led to the theory that parts of the striatum collate information from different cortical and subcortical inputs before passing that information along to adjacent striatum and ultimately the thalamus in integrative CSTC ‘spirals’ rather than distinct ‘loop’ patterns (Milad and Rauch, 2012). Along with this change, the dACC, classically thought to be involved in error processing and fear expression, has become an important focus in the pathophysiology of OCD. Given its significant connectivity with surrounding frontal cortical and subcortical regions, as well as its role in decision-making (Sheth et al., 2012), the control signal theory places the dACC at the center of limbic, associative and executive integration as a consolidative hub for determining and executing behavioral responses to environmental stimuli (McGovern and Sheth, 2017). This theory suggests that maladaptive behaviors typical of OCD may be a result of a pathologically active dACC, leading to continued action in response to an environmental stimulus regardless of the actual presence of that stimulus.

A broad network of cortical and subcortical regions plays a role in the complex behavioral phenotype of OCD. Continued investigations into the pathophysiology of OCD help to shape our understanding of how neuromodulation alleviates symptoms and plays a large role in defining optimal stimulation targets and settings.

## Deep Brain Stimulation (DBS) for OCD – Target Evolution

Initially DBS for OCD targeted the entire dorso-ventral length of the anterior limb of the internal capsule (ALIC), in which fibers travel between prefrontal cortex and the deep nuclei of the thalamus and striatum (Nuttin et al., 1999, 2003). These initial studies from Belgium used quadripolar electrodes with 3 mm contacts spaced 4 mm apart, with the deepest contact located near the border of or within the nucleus accumbens (NAc) and ventral striatum (VS), anterior to the anterior commissure. Four of the 6 patients underwent alternating sham or active stimulation for 3 months. Of these 4 patients, 3 met criteria for response, with an average reduction of YBOCS score from 32.3 with sham stimulation to 19.8 with active stimulation. More equivocal results were seen another study, which contained a blinded, sham-controlled phase with alternating 3-week on-off periods and a 4–23 month un-blinded open-label follow-up phase (Abelson et al., 2005). In the blinded phase, one of four met criteria for response with 67% improvement from baseline. In the open-label phase, two of the four patients achieved a 44% and 73% reduction in Y-BOCS compared to baseline.

A subsequent multi-center, open-label study utilized more posterior stimulation sites, specifically targeting the junction of the anterior commissure and the ventral capsule (VC), with the most distal contact extending into the VS (Greenberg et al., 2006). Ten patients were initially recruited, and eight were followed to the clinical endpoint of 36 months. Whereas only 1 of 10 patients demonstrated ≥35% reduction in Y-BOCS at 1 month, 4 of 8 patients did so at 36 months. Co-morbid depression and anxiety as well as global functioning also improved significantly.

Another study examined VC/VS DBS in six OCD patients using a blinded, staggered onset design (Goodman et al., 2010). Three patients were randomized to active DBS at 1 month after implantation, whereas the other three patients were randomized to active DBS at 2 months. The treatment effect was analyzed in a double-blinded fashion from months 1–3 after implantation, after which the study switched to an open-label phase. Although there appeared to be a reduction in Y-BOCS only after active DBS was started, there were no statistically significant differences between the two groups, likely due to short duration of the double-blinded period. Nonetheless, at 12 month follow-up, four out of the six patients experienced greater than ≥35% decrease in Y-BOCS severity. Three out of the four responders reached the criteria during the double-blind phase, whereas the fourth required activation of a second monopolar contact and increase in stimulation voltage at month 8. Interestingly, co-morbid depression (present in all six patients) improved significantly as a whole, and two of the three non-responders requested that stimulation be continued due to the perceived improvement in their mood symptoms.

Long term outcomes from initial studies (Nuttin et al., 2003; Greenberg et al., 2006) were published in a multi-institutional study from four institutions in the United States and Europe, reporting the results of 26 patients implanted at VC/VS with outcomes up to 36 months (Greenberg et al., 2010). Importantly, this group noted differences in efficacy between cohorts of patients implanted early in the study, and those implanted later in the study. They determined that as the study progressed, the target moved posteriorly, approaching the intersection of the ventral capsule and anterior commissure. Furthermore, they noted that this more posterior targeting allowed clinical improvement with lower stimulation amplitudes, suggesting proximity to the true target. The outcomes from this study led to the 2009 FDA approval of ALIC DBS for OCD under a Humanitarian Device Exemption (HDE).

In a 2010 study from Netherlands, the first to use a double blind, sham-controlled design, the authors targeted the NAc 4 mm deep to the commissural plane using an electrode with tightly spaced contacts (1.5 mm length with 0.5 mm spacing). The authors reported a 46% reduction in Y-BOCS in 16 patients, and the double-blind two week cross-over study of active versus sham stimulation confirmed the treatment effect (Denys et al., 2010).

BNST stimulation came to the forefront after a study of 24 patients who underwent DBS for intractable OCD in Belgium (Luyten et al., 2016). The study was designed first with an open label optimization period in which stimulator settings could be freely modified by psychiatrists to optimize settings over several months. After optimization of stimulation settings and determination of response (>35% YBOCS reduction), subjects were randomized into a double-blind crossover withdrawal period with two arms. One arm underwent 3 months of stimulation followed by 3 months without stimulation (ON-OFF), and the other arm underwent 3 months without stimulation followed by 3 months with stimulation (OFF-ON). After this double-blind crossover period, subjects were unblinded and allowed to choose if they would like to continue stimulation. In a *post hoc* analysis, patients were subdivided into those having active contacts primarily in the ALIC (6 patients), those having active contacts near the BNST (15 patients), and those having comparable stimulation of both areas (3 patients). One out of six ALIC-stimulated patients showed a clinical response (>35% decrease in Y-BOCS score), while twelve out of fifteen BNST-stimulated patients showed favorable outcome. These outcome differences could not be explained by differences in stimulation parameters, leading the authors to conclude that the BNST may be the favored target for electrode placement in the treatment of OCD. The investigators achieved 67% response rate at randomization and 83% response rate at last followup in subjects whose stimulation included the BNST. This excellent outcome was likely aided by smart trial design allowing for individualized stimulation optimization followed by blinded sham-controlled stimulation. The electrode trajectory comparison of ALIC-only versus BNST stimulation in this study was pivotal in highlighting the BNST as a target for improving clinical outcomes (Luyten et al., 2016; Raymaekers et al., 2017).

The BNST is thought to be important for control of anxiety, stress, and compulsion. A study of selective optogenetic stimulation of excitatory or inhibitory BNST inputs to the ventral tegmental area produced anxiogenic or anxiolytic behavior, respectively (Jennings et al., 2013), and stimulation of excitatory BNST inputs to the NAc facilitated reward-seeking behavior in rodent models (Jennings et al., 2013).

**FIGURE 3:**
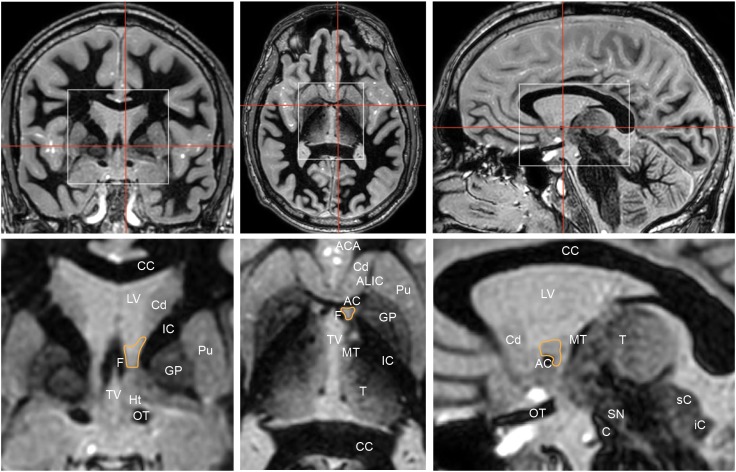
Coronal (left), axial (middle), and sagittal (right) Fast Gray Matter Acquisition T1 Inversion Recovery (FGATIR) sequences highlight the position of the bed nucleus of the stria terminalis (orange outline). Red lines in top row indicate corresponding cuts in other axes. White boxes in top row show regions that are illustrated in greater detail below. Coronal cut is 1 mm posterior to the posterior aspect of the anterior commissure. The bed nucleus of the stria terminalis is bounded laterally by the internal capsule, medially by the fornix. AC, anterior commissure; ACA, anterior cerebral arteries; ALIC, anterior limb of internal capsule; C, crus cerebri; CC, corpus callosum; Cd, head of caudate; F, column of fornix; GP, globus pallidus; Ht, hypothalamus; IC, internal capsule; iC, inferior colliculus; LV, lateral ventricle; MT, mammillothalamic tract; OT, optic tract; Pu, putamen; sC, superior colliculus; SN, substantia nigra; T, thalamus; TV, third ventricle.

The BNST is comprised of medial (BSTM) lateral (BSTL), central (BSTC) components. On human MRI imaging, the BST is bounded laterally by the internal capsule and medially by the fornix (Figure 3). On coronal MRI, the posterior border of the BNST resides anterior to the interventricular foramen of Monroe, and the anterior border lies just anterior to the crossing fibers of the anterior commissure (Theiss et al., 2017). The radiographically defined ventral border of the BNST is the superior aspect of the anterior commissure, and the superiorly, the BNST is bounded by the ventral border of the caudate.

**FIGURE 4:**
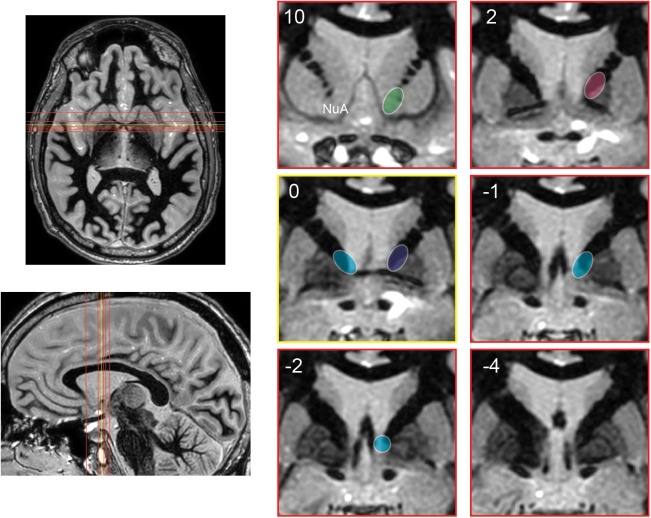
Schematic representation of the evolution of stimulation targets posteriorly over time. Axial and sagittal images depict position of coronal slices (red) relative to anterior commissure (yellow). Coronal images are shown at 10 and 2 mm anterior to the anterior commissure, at the anterior commissure, and 1, 2, and 4 mm posterior to the anterior commissure. Approximate stimulation targets are shown for various studies, emphasizing the posterior progression of stimulation targets. Nuttin et al. (1999, 2003) (green). Greenberg et al. (2010): early cohort (pink), late cohort (blue). Luyten et al. (2016) and Raymaekers et al. (2017): targets with greatest stimulation effect (teal). NuA, nucleus accumbens.

It is likely not just stimulation of the BNST that has led to better outcomes over the original ALIC target. By shifting the target posteriorly to the BNST, stimulation is applied to a wider subset of adjacent traversing white matter tracts in the ventral capsule, therefore engaging a wider cortical/subcortical network (Figure 4). In fact with BNST targeting, the therapeutic contact is often superficial to the BNST among white matter tracts rather than in a grey matter nucleus (van den Munckhof et al., 2013).

After implantation, the optimal stimulation parameters are determined empirically. In general, frequency and pulse width are held constant, while voltage is changed at each contact. Contacts with lowest threshold voltage for achieving a beneficial effect with a tolerable side effect profile are chosen (Morishita et al., 2014). In ALIC or VC/VS DBS, best combination of therapeutic benefit and tolerability was achieved with more ventral contacts. However, at higher voltages, stimulation of ventral contacts produced alteration in taste and smell as well as autonomic changes including increased breathing rate, sweating, heat sensation, and fear (Okun et al., 2006). Most recently, in a small study of 3 patients, authors sought to describe the differential effects of stimulating the ALIC versus the BNST, versus combined ALIC/BNST stimulation (Winter et al., 2017). The surgical target for this study was 2 mm posterior to the anterior commissure at the level of the commissural plane. The authors describe that high voltage stimulation of the BNST or ALIC/BNST led to increased levels of anxiety, tension and discomfort. However, lower-amplitude stimulation (1 and 2V) of all target regions led to improvement in symptoms.

## Future Directions

The optimal target for DBS in OCD remains unclear, with comparable efficacy observed across different stimulation sites. Indeed even the concept of the optimal target has evolved, from thinking of the target as a gray matter structure to conceiving of the target as a white matter bundle that connects to several regions of the symptomatic network. More recent studies have focused on detailing the precise positioning and trajectory of electrodes across individual patients, and such efforts have yielded valuable insights including the importance of BNST (Morishita et al., 2014; Luyten et al., 2016). While randomized controlled trials have provided valuable insights into treatment effect and electrode trajectory, a multi-institutional registry is needed to categorize patient demographics, co-morbidities, post-operative electrode contact positioning and trajectory, programming parameters, and outcome. Indeed, similar efforts are currently being directed at DBS for Tourette syndrome (Deeb et al., 2016).

Significant focus has been placed on defining optimal fiber bundles for targeting, rather than specific anatomical grey matter nuclei. This can be achieved by using high-resolution diffusion tractography to visualize relevant CSTC tracts and allow for more tailored stimulation based upon individual connectomes. One such study has found that there is considerable individual heterogeneity in the OFC-thalamic circuitry within VC/VS. Stimulation of different VC/VS contacts can alternatively stimulate medial and/or lateral orbitofrontothalamic fibers, which are functionally distinct (Makris et al., 2016). These methods can also be combined with electric field modeling to characterize the theoretical DBS voltage distribution in brain tissue. In this manner, activated axon fiber bundles adjacent to the electrode as well as the grey matter areas activated by these axon fiber bundles were identified and correlated with clinical outcome in patients who had undergone ALIC DBS for OCD. Importantly, modulation of the dorsolateral prefrontal cortex correlated with excellent clinical outcome, whereas high activation in the lateral OFC was associated with clinical non-responders (Hartmann et al., 2015).

These structural and modeling studies do not reveal any underlying electrophysiological or functional changes that mediate the clinical effects of DBS for OCD. In order to address these questions, functional MRI studies have been performed during a reward-anticipation task and during resting state in patients who had undergone bilateral NAc DBS for OCD for at least 12 months (Figee et al., 2013). When DBS stimulation was turned off, NAc activity in patients was lower than controls during the reward anticipation task, whereas the NAc activity returned to normal after DBS stimulation was turned on. Furthermore, excessive resting state frontostriatal connectivity in OCD patients returned to baseline after DBS stimulation was turned on, and the extent of this reduction in connectivity correlated with improvement in Y-BOCS. Therefore, pre-operative screening studies using functional MRI may be a valuable adjunct to assess individual CSTC circuitry in OCD patients and choose the target for DBS accordingly.

The studies described above have used a variety of trial designs, from randomized staggered onset to up-front randomization to open-label optimization followed by double-blind discontinuation. Each has its advantages and disadvantages, and future work will need to consider these carefully. Not only are trials very expensive, but improper consideration of contributing factors can stack the odds against seeing a therapy-related effect. For example, two recent industry-sponsored trials of DBS for depression were discontinued at interim analyses due to concern for insufficient difference between active and sham stimulation. The RECLAIM (Dougherty et al., 2015) and BROADEN (Holtzheimer et al., 2017) trials both used up-front randomization designs. Critical analysis of this experience has identified several features that should be considered in future designs. For example, up-front randomization may be more susceptible to placebo effect (Bari et al., 2018). In addition, too short a blinded interval, especially when restricting exploration of the stimulation parameter space, may prevent differentiation between sham and active stimulation.

Intraoperative local field potentials (LFPs) recorded from microelectrodes reflect aggregate activity of a population of neurons, which can be separated into distinct frequency bands based on the frequency of oscillations. They have been shown in other CSTC-based disorders such as Parkinson’s disease to correlate with symptoms and are potentially useful in predicting ideal electrode position in STN DBS (Neumann et al., 2016; Telkes et al., 2016). However, precise LFP patterns that characterize OCD have yet to be defined. Nonetheless, recordings from BNST in rodent models of OCD have demonstrated characteristic increase in power of delta (1–4 Hz) and gamma (30–45 Hz) oscillations during the initiation of compulsive behavior, whereas beta (12–30 Hz) oscillations increased after cessation of compulsive behavior (Wu et al., 2016). Importantly, the magnitude of these changes correlated with the efficacy of BNST stimulation in suppressing the compulsive behaviors.

Few published studies have examined LFPs during DBS for OCD in human patients (Neumann et al., 2014). While no characteristic LFP changes were found in OCD patients that correlated with symptoms, response to stimulation or triggered compulsions were not measured. Two projects funded by the NIH Brain Research through Advancing Innovative Neurotechnologies (BRAIN) Initiative are using such an approach to help identify physiological biomarkers that can aid in programming and pave the way for closed-loop stimulation (NCT03457675, NCT03184454).

Finally, DBS is a valuable investigative tool to examine the pathogenesis of OCD, which is a clinically heterogeneous disorder characterized by diverse clinical endotypes. Interestingly, alterations in distinct neural systems underlie stereotypical behaviors such as washing, checking, and hoarding (Mataix-Cols et al., 2004). Furthermore, several neuropsychological and imaging studies have demonstrated broad deficits in executive function in OCD in domains such as impulsivity, attention, and decision making (Gu et al., 2008; Vaghi et al., 2017). Segregating OCD patients based on symptomatology and assessing their response to DBS will provide valuable insights into how stimulation of ALIC, VC/VS, and BNST circuits impact varied manifestations of OCD. For instance, a recent study demonstrated that patients who have successfully undergone ALIC DBS nonetheless exhibit persistent deficit in impulse control and decision-making on formal neuropsychological testing, suggesting that these aspects of executive function may be mediated by a separate circuit other than the orbitofrontal loop targeted by DBS (Grassi et al., 2018). Rather than the current empiric approach to DBS programming, these data may ultimately allow for more selective, algorithmic selection of optimal stimulation parameters.

## Conclusion

Deep brain stimulation targets for OCD have evolved significantly over time, both in anatomic location and concept. It is now clear that targeting posteriorly and ventrally within the capsule leads to improved outcomes. The overall trajectory of leads is also clearly important, as therapeutic effect is likely derived from direct stimulation of white matter tracts that lead to modulation of broad cortical and subcortical networks. Despite significant advance, the underlying alterations in the CSTC circuitry that mediate improved clinical efficacy remain unclear, the optimal stimulation site is still not precisely defined, and trial designs continue to be debated. Nevertheless, improved anatomical and functional models of the involved regions, along with an infusion of technology, has led to promising new lines of study. Given the severity of this disorder, investigators will continue improving DBS for OCD for years to come.

## Author Contributions

AV and SS were responsible for conception of the article and critical review. SL and PK were co-first authors responsible for literature search and primary writing of the manuscript. JJ-S and WG provided critical review of the manuscript.

## Conflict of Interest Statement

The authors declare that the research was conducted in the absence of any commercial or financial relationships that could be construed as a potential conflict of interest.
